# Efficiency of mild ovarian stimulation with letrozole co-treatment in expected low ovarian responders

**DOI:** 10.5935/1518-0557.20250161

**Published:** 2026

**Authors:** Alfredo Cortés-Vazquez, Ismael Perez-Bautista, J. Andreas Mejicanos-Quiñonez, Hector R. Salazar-Ochoa, M. Carmen Peraza-Briones, J. Daniel Moreno-García, Alfredo L. Cortés-Algara

**Affiliations:** 1 Reproductive Endocrinology Department, Centro Médico Nacional 20 de Noviembre, Mexico City, Mexico

**Keywords:** efficiency, ovarian, mild, letrozole, stimulation

## Abstract

**Objective:**

Ovarian stimulation (OS) is a critical step in assisted reproductive techniques (ART); it has been routinely adopted since it substantially increases the whole process efficiency. Our study aims to compare the efficiency of mild OS with letrozole co-treatment vs. conventional OS in expected low ovarian responders.

**Methods:**

A single-centre, observational, comparative, non-experimental and retrospective study using our database at the Reproductive Endocrinology Department at Centro Médico Nacional 20 de Noviembre in Mexico City was performed.

**Results:**

We compared the total rFSH dosage per oocyte, we noticed a statistically significant difference between groups, Group A 388.9 vs. Group B 667.2IU per retrieved oocyte (*p*<0.0002, 105 to 450 IU CI 95%), Comparing total rFSH dosage per follicle, we noticed a statistically significant difference among Group A 397.5 vs. Group B 590.2IU per follicle (*p*<0.014, 39.2 to 346.2 IU, CI 95%).

**Conclusions:**

In expected poor ovarian responders, mild ovarian stimulation with letrozole co-treatment is much more efficient than conventional stimulation.

## INTRODUCTION

Since 1980, in vitro fertilization (IVF) has allowed millions of couples to fulfil their dream of parenthood (Edwards *et al.*, 1980). Ovarian stimulation (OS) is a critical step in assisted reproductive techniques (ART); it has been routinely adopted since it substantially increases the whole process’s efficiency. However, some conditions may be challenging, particularly low ovarian response. Couples facing a low ovarian response usually face multiple cycles, costly procedures and IVF failure. As described by our group, the low ovarian response can affect as much as 29% of cycles in a Mexican IVF program (Cortés-Vazquez *et al.*, 2024). Various ovarian stimulation protocols have been described to improve pregnancy outcomes in these patients. Unfortunately, none of these protocols has been demonstrated to be superior (Pandian *et al.*, 2010; Sood *et al.*, 2021; Naik *et al.*, 2024). Mild ovarian stimulation for IVF is defined as a protocol in which the ovaries are stimulated with gonadotropins, other pharmacological compounds, or both to limit the number of oocytes after stimulation for IVF’ according to the International Glossary on Infertility and Fertility Care by the International Committee for Monitoring Assisted Reproductive Technology (Zegers-Hochschild *et al.*, 2009). Evidence is clear and has shown that mild ovarian stimulation with co-treatment by oral compounds is as effective as high doses of gonadotropins in IVF cycles, with lower duration of ovarian stimulation, lower amount of gonadotropins used and comparable to conventional ovarian stimulation in terms of the ongoing pregnancy rates (Youssef *et al.*, 2017; Datta *et al.*, 2021). Other benefits include reducing complexity and patient discomfort, enabling patients to continue treatment to increase their chances of parenthood. To our knowledge, no study has compared the efficiency of mild OS with letrozole co-treatment vs. conventional OS in expected low-ovarian responders. Our study aims to compare the efficiency of mild OS with letrozole co-treatment vs conventional OS in expected low ovarian responders.

## MATERIAL AND METHODS

### Setting, size and study design

We performed a single-centre, observational, comparative, non-experimental and retrospective study using our database at the Reproductive Endocrinology Department at Centro Médico Nacional 20 de Noviembre in Mexico City. Notably, the Ethics Committee (Institutional Board Review) from Centro Médico Nacional 20 de Noviembre approved this study (Registry Number 291.2024) and was conducted in a public IVF centre from January 1st 2020 to December 31st 2023. Patients were eligible if they had the following criteria: i) first autologous IVF intended for fresh embryo transfer; ii) women aged between 25 to 45 years old with an antral follicle count (AFC) equal to or less than seven follicles received a conventional or a mild ovarian stimulation with letrozole co-treatment. A mild ovarian stimulation protocol was defined as a mean daily dose of <150 IU gonadotropin on a gonadotropin-releasing hormone (GnRH) antagonist cycle. Exclusion criteria were clomiphene co-treatment, natural cycle, progestin-primed ovarian stimulation, pretreatment with oral contraceptive pills or adjuvant treatment, fertility preservation for oncologic patients and cancelled or incomplete cycles. A non-probabilistic convenience sampling method was performed. Patients with low ovarian reserve were grouped according to the ovarian stimulation protocol received. Group A received mild ovarian stimulation with letrozole co-treatment, while Group B received conventional ovarian stimulation. Ovarian stimulation applied in Group A was a fixed protocol of 5 mg letrozole for 5 days, orally, and 150 IU of recombinant FSH (rFSH, Gonal F, Merck-Serono) subcutaneously, from stimulation day 3 or 5 until trigger day. Stimulation was cancelled if there was no follicular development on stimulation day 8.

Meanwhile, Group B was stimulated with 225-450 IU rFSH plus 150 IU recombinant LH (Luveris, Merck-Serono) from cycle days 2 to 3. A GnRH antagonist (Cetrorelix, Cetrotide, Merck-Serono) was initiated on stimulation day 6 or when a follicular diameter reached 12 to 14mm. Oocyte retrieval was performed 34 to 36 hours after administering human chorionic gonadotropin (Ovidrel, 250mcg, Merck-Serono). In all cases, an intracytoplasmic sperm injection (ICSI) was performed. The luteal phase was supported by intravaginal progesterone capsules (Geslutin, 200mg, Asofarma, Mexico) three times daily from oocyte retrieval day to until pregnancy test day or, in the event of conception, until the tenth week of pregnancy. Embryos were scored using the British Fertility Society and Association of Clinical Embryology scoring systems for cleavage-stage embryos (Cutting *et al.*, 2008); grade 1 to grade 3 embryos were transferred. The blastocyst-stage embryos were scored according to the grading system described by Gardner & Schoolcraft (1999); AA, AB, BA, BB, BC, CA and CB were considered suitable for transfer. As a general policy, an elective single embryo transfer is performed in women <35 years of age and double embryo transfer is offered to women beyond 35 years of age. Young women or those with three embryos usually had a single blastocyst transfer. Surplus embryos were cryopreserved by vitrification for future transfer with either natural or hormone replacement therapy cycles. A pregnancy test by estimating serum HCG was performed to confirm pregnancy. In the event of a positive pregnancy test, viability and location of the pregnancy were confirmed by a transvaginal ultrasound scan at or beyond 6 weeks of gestation.

### Variables

Data obtained from our database included age, body mass index (BMI), AFC, stimulation length, total rFSH dose, number of retrieved and mature oocytes, maturation rate, follicles >12mm on trigger day, total gonadotropin dose, and clinical pregnancy rate. The total rFSH dose per oocyte, total rFSH dose per mature oocyte, and total rFSH dose per follicle were calculated for each patient.

### Data sources

Data was obtained from our department’s database. All records were reviewed according to the Federal Transparency & Access to Public Information Act Law. We also committed to following the Personal Data Protection General Law. Incomplete information on the database was an elimination criterion for our study.

### Outcomes

The primary outcomes were to compare the efficiency, as defined by the total rFSH dose per oocyte, total rFSH dose per mature oocyte, and total rFSH per follicle, between mild and conventional ovarian stimulation in expected poor ovarian responders. The secondary outcome was to compare the clinical pregnancy rates between mild and conventional ovarian stimulation.

### Statistical Methods

Data are presented as total number, mean±Standard Deviation, and percentages. A Kolmogorov-Smirnov test was performed to evaluate the data for normality. Subsequently, comparisons were performed using the Student’s t Test. We calculated the clinical pregnancy rate based on the presence of at least one fetal heartbeat per embryo transfer.

## RESULTS

Between January 2020 and December 2023, we included 176 couples. Our population had a mean age 36.6(±3.5) years old, 25.2(± 4.7) kg/m^2^ BMI, 5.0(±1.7) AFC, 1872.48(±872.5) IU total rFSH dosage, 9.3 (±2.5) stimulation days, 4.2(±3.5) retrieved oocytes, 2.7(±2.6) mature oocytes, 561.2(±577.7) total IU rFSH dosage per oocyte, 648.4(± 661.3) total IU dosage per mature oocyte, 516. (±508) total IU rFSH dosage per follicle as can be seen at Table 1. Baseline characteristics between the two groups were similar in both (Table 2). A Kolmogorov-Smirnov test was performed to verify data normalcy for each variable. After classifying patients according to their ovarian stimulation, Group A had 67 patients who received mild ovarian stimulation, while Group B had 109 and received conventional ovarian stimulation.

**Table 1:** Clinical data and outcomes.

	Mean (n=176)	SD
**Age (years)**	36.6	3.5
**BMI (kg/m^2^)**	25.2	4.7
**AFC**	5.0	1.7
**Stimulation length (days)**	9.3	2.5
**Total rFSH dosage (IU)**	1872.4	872.5
# **Oocytes retrieved**	4.2	3.5
# **Mature oocytes retrieved**	2.7	2.6
# **Follicles >12 mm**	5.0	3.3
**Maturation rate (%)**	58.0	35.9
**Clinical pregnancy rate (%)**	15.8	1.5
**Total rFSH dosage / oocyte**	561.2	577.7
**Total rFSH dosage/mature oocyte**	648.4	661.3
**Total rFSH dosage/ follicle**	516.8	508.3

Data are presented as total number, mean± standard deviation (SD or number).

**Table 2:** Comparison between the two groups .

	Group A (n=67)	Group B (n=109)	p value
**Age (years)**	37.1 (±3.4)	36.2 (±3.5)	NS
**BMI (kg/m^2^)**	25.0 (±5.8)	25.3 (±3.9)	NS
**AFC**	5.1 (±1.9)	5.0 (±1.5)	NS
**Stimulation length (days)**	9.5 (±2.9)	9.2 (±2.3)	NS
**Total rFSH dosage (IU)**	1126.8 (±509.8)	2362.2 (±696.7)	0.000
# **Oocytes retrieved**	3.1 (±2.7)	4.8 (±3.8)	0.002
# **Mature oocytes retrieved**	2.2 (±2.1)	3.0 (±2.9)	0.045
# **Follicles >12 mm**	4.0 (±2.5)	5.7 (±3.6)	NS
**Maturation rate (%)**	58.5 (±37.6)	57.7 (±35.0)	NS
**Clinical pregnancy rate (%)**	16.6 (±2.1)	15 (±1.9)	NS
**Total rFSH dosage / oocyte**	388.9 (±349.9)	667.2 (±660.4)	0.000
**Total rFSH dosage/ mature oocyte**	440.0 (±413.6)	776.5(±748.7)	0.000
**Total rFSH dosage/ follicle**	397.5 (±392.0)	590.2 (±557.1)	0.001

NS= non significant.

**Figure 1:**
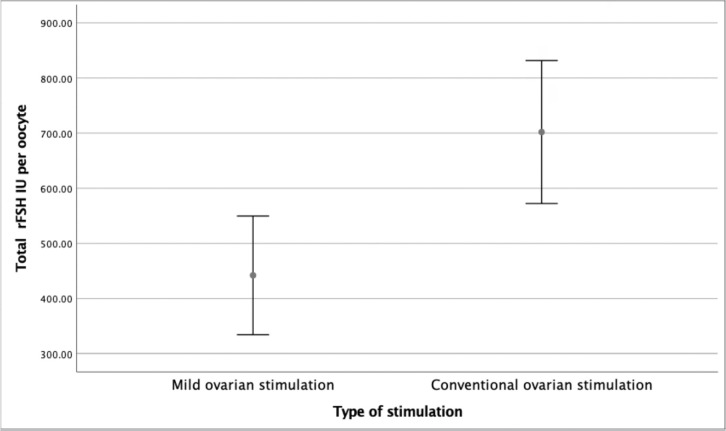
Total FSH IU per oocyte X Type of Stimulation.

**Figure 2:**
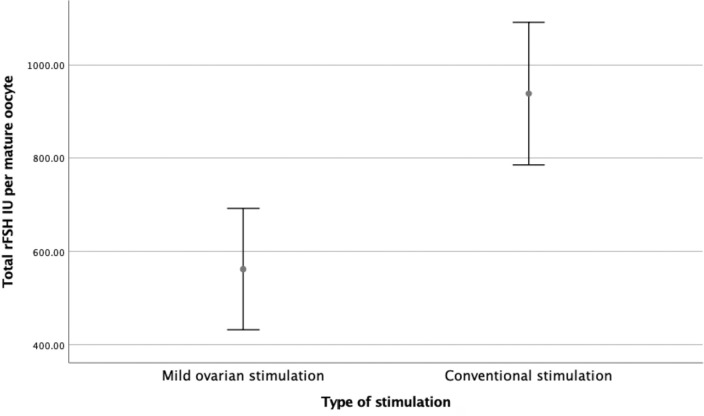
Total FSH IU per mature oocyte X Type of Stimulation.

**Figure 3:**
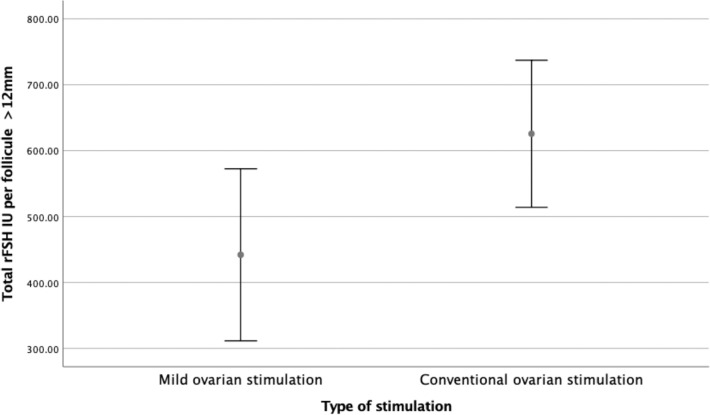
Total FSH IU per follicule X Type of Stimulation.

When we compared the total rFSH dosage per oocyte, we noticed a statistically significant difference between groups, Group A 388.9 *vs*. Group B 667.2 IU per retrieved oocyte (*p*<0.0002, 105 to 450 IU CI 95%), as can be seen in Figure 1. Total rFSH dosage per mature oocyte was statistically significant, with Group A 440.0 *vs*. Group B 776.5 IU per mature oocyte (*p*<0.000, 139.6 to 533.4 IU, CI 95%), as shown in Figure 2. Comparing total rFSH dosage per follicle, we noticed a statistically significant difference among Group A 397.5 *vs*. Group B 590.2 IU per follicle (*p*<0.014, 39.2 to 346.2IU, CI 95%) as shown in Figure 3. Meanwhile, stimulation days did not significantly differ between both groups, Group A 9.5 *vs*. Group 9.2 days (*p*=0.545, 0.5 to 1.0, CI). When we compared pregnancy rates among groups, they were comparable, Group A 16.6 *vs*. 15.0 (*p*=0.677, -3.5 to 15.3, CI).

## DISCUSSION

Our study shows that mild stimulation with letrozole co-treatment is more efficient than conventional ovarian stimulation in patients with an expected low ovarian response, requiring lower total rFSH dosage per oocyte retrieved (388.9 *vs*. 667.2IU, *p*<0.0002), lower total rFSH dosage per mature oocyte (440.0 *vs*. 776.5 IU, *p*<0.000) and lower total rFSH dosage per follicle (397.5 *vs*. 590.2 IU, *p*<0.014). Even though conventional ovarian stimulation has a significantly higher number of retrieved oocytes (3.1 *vs*. 4.8, *p*<0.002), there were no major differences in terms of clinical pregnancy rates (16.6 *vs*. 15.0%, *p*=0.667). Other authors have similar conclusions, showing a lower total amount of gonadotropins, fewer retrieved oocytes, fewer mature oocytes and comparable ongoing pregnancy rates (Youssef *et al.*, 2017). Datta *et al.* (2021) showed through a meta-analysis that mild ovarian stimulation in patients with a diagnosed poor ovarian response is as effective as conventional ovarian stimulation in terms of live birth rate. The fact that pregnancy rates are not different between groups, even when mild ovarian stimulation had fewer oocytes, could be the effect of intense ovarian stimulation on oocyte development. In a prospective randomized controlled trial, Baart *et al.* (2007) showed that even doses such as 225 UI FSH are associated with a higher proportion of aneuploid and mosaic embryos than mild ovarian stimulation. However, Irani *et al.* (2020) found no influence of total gonadotropin dosage and duration of ovarian stimulation on aneuploidy rates or euploid embryos.

Our findings are consistent with the current evidence; one of the strengths of our study is the statistical management of our data. However, our results have some limitations. The retrospective nature of our study and small sample size may not exclude bias. Clinicians must interpret our results carefully. Since our data shows a better efficiency of mild-ovarian stimulation with letrozole co-treatment compared to conventional ovarian stimulation, a multi-centre study may help to prove the generalizability of our single-centre data.

## CONCLUSIONS

In expected poor ovarian responders, mild ovarian stimulation with letrozole co-treatment is much more efficient than conventional stimulation.
